# Early breastfeeding experiences of adolescent mothers: a qualitative prospective study

**DOI:** 10.1186/1746-4358-7-13

**Published:** 2012-09-29

**Authors:** Paige Hall Smith, Sheryl L Coley, Miriam H Labbok, Susan Cupito, Eva Nwokah

**Affiliations:** 1 Center for Women’s Health and Wellness, University of North Carolina at Greensboro, Greensboro, NC, USA; 2 Department of Public Health Education, University of North Carolina at Greensboro, Greensboro, NC, USA; 3 Carolina Global Breastfeeding Institute, Department of Maternal and Child Health, UNC Gillings School of Global Public Health, University of North Carolina at Chapel Hill, Chapel Hill, NC, USA; 4 Teen Parent Mentoring Program, Greensboro YWCA, Greensboro, NC, USA; 5 Department of Communication and Learning Disorders, Our Lady of the Lake University, San Antonio, TX, USA

**Keywords:** Breastfeeding, Adolescent health, Breastfeeding education, Preconception

## Abstract

**Background:**

Teen mothers face many challenges to successful breastfeeding and are less likely to breastfeed than any other population group in the U.S. Few studies have investigated this population; all prior studies are cross-sectional and collect breastfeeding data retrospectively. The purpose of our qualitative prospective study was to understand the factors that contribute to the breastfeeding decisions and practices of teen mothers.

**Methods:**

This prospective study took place from January through December 2009 in Greensboro, North Carolina in the U.S. We followed the cohort from pregnancy until two weeks after they ceased all breastfeeding and milk expression. We conducted semi-structured interviews at baseline and follow-up, and tracked infant feeding weekly by phone. We analyzed the data to create individual life and breastfeeding journeys and then identified themes that cut across the individual journeys.

**Results:**

Four of the five teenagers breastfed at the breast for nine days: in contrast, one teen breastfed exclusively for five months. Milk expression by pumping was associated with significantly longer provision of human milk. Breastfeeding practices and cessation were closely connected with their experiences as new mothers in the context of ongoing multiple roles, complex living situations, youth and dependency, and poor knowledge of the fundamentals of breastfeeding and infant development. Breastfeeding cessation was influenced by inadequate breastfeeding skill, physically unpleasant and painful early experiences they were unprepared to manage, and inadequate health care response to real problems.

**Conclusions:**

Continued breastfeeding depends on a complex interplay of multiple factors, including having made an informed choice and having the skills, support and experiences needed to sustain the belief that breastfeeding is the best choice for them and their baby given their life situation. Teenagers in the US context need to have a positive early breastfeeding experience, be able to identify and claim a reliable support system supportive of breastfeeding, and gain through their experience, a belief in their own agency and competency as mothers.

## Background

The breastfeeding rate among adolescent mothers in the United States is low and has been dropping since 2003. Young women are less likely to breastfeed than older mothers and have a more rapid discontinuation rate [1-3]. For these teen mothers, as with their older counterparts, knowledge of the benefits of breastfeeding is not sufficient to result in breastfeeding [4-6]. Teen mothers in the US face many challenges to successful breastfeeding that are unique to their age and situation including: coping with the stigma and embarrassment related to being a teen mother; lack of parenting readiness; need for peer acceptance; and dependence on social support systems that may not be supportive of breastfeeding [7]. A significant number of teen mothers have a low income and there is a strong association between living in poverty, crime, poor educational opportunities, teen pregnancy and low breastfeeding [1,3,8-10].

For most teens, their negative views of breastfeeding outweigh the positive. Their attitudes regarding the perceived relationship between breastfeeding and mother-infant bonding is an interesting case in point. An ethnographic study with African-American and Latina adolescent mothers found that bonding was among the better known “benefits” of breastfeeding; however, it was not uniformly perceived as a benefit [11]. This study, among others, found that “bonding” will make it more difficult for them to leave their babies with others, thus making their lives more difficult and complicate their return to school [12,13].

Many teen mothers do not have the skills to incorporate breastfeeding or milk expression into their lifestyle as students and/or employees [5,6,11,12]. This stems, in part, from their lack of knowledge of, and/or discomfort with, milk expression by pump or hand; not having a private place to express their milk or feed their baby at school or other places; and not having the skills to prevent or manage common problems like pain or leaking [6,11,13,14].

Social support from family, friends and partners are among the most important factors affecting young mothers’ infant feeding choices, yet most do not receive this support, and many are encouraged to bottle- and formula feed by family members [5,11-19]. Continuity of care and support by the health care system, those skilled in lactation, and the school system are also important [16,18,19]. Young women who received support and instruction in breastfeeding and pumping from healthcare professionals or the Special Supplemental Nutrition Program for Women, Infants, and Children (WIC) were not only more likely to begin breastfeeding, but were also more likely to maintain breastfeeding after hospital discharge [4-6,11,14,15,18,20]. Many studies indicate that adolescents who choose formula feeding did so because they thought breastfeeding would make it more difficult to return to school or work, and that the school’s lack of support, limited space for pumping, lack of time to breastfeed or pump throughout the day, and lack of on-site childcare all make breastfeeding more difficult [5,11,12,20]. These prior studies suggest that adolescent mothers, as is the situation with older mothers, have multiple role obligations in addition to their maternal role. The aim of our prospective, qualitative study was to examine how adolescent mothers’ lives and experiences shape their breastfeeding practice over time.

## Methods

### Study population and enrollment

This prospective study took place from January through December 2009 in Greensboro, North Carolina in the U.S. This study comprises part of a larger study to gather information for the development of a breastfeeding education program for teen mothers. The sample was drawn from 17 pregnant teens enrolled in a seven-week childbirth education program offered by the Young Women’s Christian Association (YWCA)’s Teen Parent Mentoring Program (TPMP) that offers multiple services for pregnant and parenting teenagers. Pregnant teens are referred to TPMP, which has been active in the community for 28 years, by schools and community agencies. The teens typically served by TPMP live in low-income communities. Their families receive governmental assistance such as Medicaid and WIC. African-American and other ethnic minority teens comprise over 90% of this population, whereas only 50% of the pregnant teens in the county are minority. The childbirth education class offers 60–90 minutes of breastfeeding education provided primarily by lactation staff at an area hospital. TPMP staff described the study to the teens that enrolled in the target childbirth class; those who were interested were given consent forms for their guardian to sign. Subsequently, the study graduate research assistant attended group meetings and those who were interested signed up for an interview. In order to be interviewed the teens had to provide a consent form signed by their guardian and also personally assent. The Institutional Review Board at the University of North Carolina at Greensboro approved this study.

### Data collection

A trained graduate research assistant conducted a semi-structured in-person baseline interview with each teen at the beginning of the seven-week childbirth education class. After delivery, and until two weeks after they ceased all breastfeeding and pumping, a TPMP staff member called each teen weekly to track infant feeding behavior, problems, responses to problems and reasons for breastfeeding and/or pumping cessation. Following the end of weekly tracking, the graduate research assistant conducted an in-person follow-up interview.

At baseline we asked these women questions on the following topics: how they felt about becoming a mother; how they expected their life might change after the baby was born; how they expected to feed their baby; what they and others around them knew or believed about breastfeeding and infant formula; and their hopes for themselves and their baby. We also asked the young women to think about the African saying “It takes a village to raise a child”, asking them who they expected to be part of their village. At follow-up we asked the mothers questions on these topics: circumstances other than mothering that were going on in their lives (such as school, work, family responsibilities, friends, the baby’s father); their living situation; a typical week and weekend day in their life; their care-giving “village”; how their life changed after becoming a mother; the joys and struggles they experienced as a mother; how they fed their baby (why they made the feeding choices they made; their infant feeding practice (breastfeeding; problems, support, reactions of others; pumping; use of formula); and their hopes for themselves and their babies. In addition, we collected baseline quantitative data on all 17 participants in the study; questions elicited demographic characteristics and breastfeeding intention.

### Data analysis

A professional transcriptionist transcribed the interviews verbatim. Our goal with the analysis was to identify each woman’s unique breastfeeding and life journey and then to compare and contrast these journeys to identify themes that cut across participants. The data were coded into categories based on the research questions and these categories were used to develop individual breastfeeding and life journeys. A teen’s breastfeeding journey consists of the following categories: rationale for breastfeeding (baseline and follow-up interviews); breastfeeding practice (weekly tracking and follow-up interviews); breastfeeding messages and support from others (follow-up interviews); breastfeeding experiences (weekly tracking and follow-up interview) and breastfeeding cessation (weekly tracking and follow-up interview). Life journey categories included: demographic characteristics; concerns about motherhood; personal goals; role experiences; living situations. The first two authors collaborated on coding and analysis; the interviews were coded line by line multiple times to ensure that all text was correctly categorized. After coding we created two files, using Microsoft Word, for each participant: one that kept the data in the order it was presented and a second that reorganized the transcript by codes by concept. We then constructed breastfeeding and life journeys for each participant and identified themes that cut across participants. Frequent team meetings and an iterative process of checking the cases against the raw data allowed us to refine journeys and themes. The use of ellipses in the quotes indicates places where we have inserted a word/words to clarify the meaning of the text.

### Definitions used

The term breastfeeding is defined in context in the quotations. For the remainder of the article, the term breastfeeding is used for feeding at the breast, and milk expression or pumping is used to describe the expression of milk via pump [21]. None of the teens in this study used hand expression.

## Results

Seventeen teens were enrolled in the target cohort. Most (12) were African-American. Of these African-American teens, one teen was self-reported as “mixed” in being African-American and Hispanic, four of the teens were White, and one was Asian. They ranged in age from 14 to 17, and their male partners were on average two years older. In terms of breastfeeding intention, seven intended to breastfeed only and an additional four intended to breastfeed and formula feed. Only two indicated they intended to exclusively formula feed and three were not sure (data for one participant was missing).

Seven agreed to participate in the prospective study. They ranged in age from 14 to 17. One teen indicated during the baseline interview that she intended to use formula whereas six intended to breastfeed. The one teen who decided not to breastfeed at all stated that *“It don’t seem likeI can breastfeed. LikeI don’t even likedoing it.”* One teen who indicated she wanted to breastfeed dropped out of the TPMP program after the baseline interview and was lost to follow-up. The results presented here are based on the remaining five mothers who intended to breastfeed and completed the baseline and follow-up interviews and infant feeding tracking. The teen study population included three African Americans, one Asian immigrant, and one Caucasian teen; therefore this study population closely reflected the ethnic proportions of the TPMP population.

### Themes from breastfeeding journeys

#### Breastfeeding intentions and practice

Our baseline interviews provided data on the teens’ rationale for their decision to breastfeed (Table 1). The belief that “breast is best” was the key rationale behind the teens’ intentions to breastfeed, and the teens’ responses did convey that they had some idea of reasons why breastfeeding is healthier. Four of the five teens indicated that they chose breastfeeding primarily because it was “best” for the baby; many of them learned about this from their childbirth classes.

**Table 1:** Elements of the teens’ breastfeeding journeys

**Teen 1**	**Teen 2**	**Teen 3**	**Teen 4**	**Teen 5**
**DEMOGRAPHIC CHARACTERISTICS AT BASELINE**				
14-year old immigrant, 7^th^ grade, expecting her first child.	16-year old, 9^th^ grade, expecting her first child after a previous miscarriage.	17-year old, 12^th^ grade, expecting her first child.	17-year old, 12^th^ grade, expecting her first child.	16-year old, 11^th^ grade, expecting her first child.
**DEMOGRAPHIC CHARACTERISTICS AT FOLLOW-UP**				
At nine weeks postpartum she was in school and not working. She married the father.	At eight weeks postpartum she is back in school and working about 40 hours/weeks, after school and on weekends.	At four weeks postpartum she is still in the homebound program and will return to school soon. She is not working.	At six months postpartum she is in her first semester of college. She is not working.	At six weeks postpartum she is out of the school for the summer. She is not working.
**FEEDING INTENTION AT BASELINE**				
She decided to do breastfeeding and bottle feeding for the baby, and intends to breastfeed for six weeks then start formula when she returns to high school.	She wants to do both breastfeeding and formula feeding, and she is uncertain about which type of feeding to do the most.	She really wants to breastfeed, despite the negative messages that she received about breastfeeding. Should consider formula feeding only if she has to.	She plans to breastfeed and pump when she goes to her dad’s home or grandma’s home.	She plans to breastfeed as long as she can, and she will pump when someone else is taking care of the baby.
**RATIONALE FOR FEEDING INTENTIONAT BASELINE**				
Breastfeeding is best for baby’s health and her relationship with the baby	She learned in class that breastfeeding is “excellent thing”	Breastfeeding is best for baby	Her determination to breastfeed stemmed from an opposition to formula, financial constraints; need for financial independence, her mother’s positive breastfeeding experience, and the encouragement of the TPMP: *“I don’t want to go to formula. I don’t want my baby to even know what formula is…Friends I’ve had – like a friend I’ve lived with she had a baby not too long ago. And her baby was going to the hospital every other week, and were like ‘What is wrong with her’. And she’s like ‘Oh, it’s her formula is breaking her out. Oh her formula is messing up her stomach’. She had to change her formula so many times.”*	Breastfeeding is best for baby
“*I’m going to breastfeed. When I’m school, then we will do the bottle feeding… when I’m in school the baby need milk. So, I have to do the bottle feeding… I believe breastfeeding is good for baby, make them healthy, and you can also get a relationship with your baby when you breastfeed, I think.” *	*“I was learning in the classes that it’s better milk. They have more – they have less ear infections, um, less breathing problems, you know? More healthy, more routine and stuff like that… I think it’s an excellent thing because the child has better qualities.”*	“*That’s one thing that I really wanted to do was breastfeed… it is the best thing for the baby. Even if I won’t be able to breastfeed I maybe still could pump, and then – I just want her to be able to get everything that she needs.”*	*“I don’t even want to be in the state’s system. The only thing I want to be in the state system for is Medicaid…because I can’t pay for Medicaid…My main reason [for breastfeeding] was less money I had to spend. Like I said, I don’t have the funds to get formula milk. . . Two my mom did it…”*	“*I plan on breastfeeding as long as I can, but don’t know how long that’s going to be… it’s better for the baby…I mean I’m going to breastfeed no matter what, even if somebody says like I can’t do it. It’s going to make me want to do it even more.”*
		At her follow up interview she stated *“I mean her [the baby’s] father wanted me to [breastfeed]. So he pushed me to. But then I started finding out like going to the TPMP classes they tell you how much breast milk is good for her.*		
**BREASTFEEDING PRACTICE**				
Total human milk for 56 days: breastfed with pumping for 7 days; continued pumping for 51 days; introduced formula during week 6. She started formula the week she started school. At week 7 she was still pumping and using formula; the baby was receiving pumped milk 4x during day and formula 2x at night. (follow-up interview was conducted too early).	Total human milk for 4 days; no pumping; introduced formula during week 1 and used cabbage leaves to dry up milk	Total human milk for 21 days; breastfed only for 2 days; pumped and breastfed for 10 days; pumped 9 days more; introduced formula in week 2 and was exclusively formula feeding by week 4	Human milk for at least 6 months when follow-up interview was conducted; breastfed exclusively for 42 days with no pumping; started pumping after 42 days and continued both	Human milk for 28 days; breastfed only for 3 days when the nurse introduced formula during week 1. She pumped 1-2x per day until week 5.
**RATIONALE FOR BREASTFEEDING CESSATION AT FOLLOW-UP**				
She did not like putting the baby to the breast *“It feels funny, it tickles” * and *she “prefers pumping breast milk with bottle” *. She continued to pump and use formula, but she quit pumping during the day because of school: “*It is a problem for the school. Like sometime it get really, you know, wet and hurt and there’s no pump at school * [the school has a pump but she does not pump there]. *Sometimes I have to get all wet and have to cover up so people don’t see. That’s a problem.”* She indicated being tired and concerned about keeping up with school work; baby is eating a lot at night.	She stated that her *“breasts felt like hard rocks”.* She stated it was painful when milk comes in. She thought she could not do anything about the pain: “*It’s painful…for the breast to get full again. That’s like where they couldn’t do nothing about that.”* At day 4 the nurse advised her to put the baby on the bottle since the nurse thought the baby was not getting enough.	She stopped breastfeeding *“because it’s hard to be honest. She digests so quickly so hunger more, waking up in the middle of the night, too hard for me”.* She pumped between feedings using a hand pump, which was a *“pain*”. *“I have to see her and be with her always had to be on top of things all the time”.* She also did not want to pump when she went back to school *“When I go back to school and I’m still breastfeeding it’s going to be hard also, because I’m still producing milk, because I can’t relieve it how I normally do. Because, then, I would have to pump during lunch. I don’t think I could go for that long. Because when I was breastfeeding I couldn’t go for more than three hours without pumping to relieve. That’s how much would come in. And it was so bad that like my glands under my arms were filled with milk. And it was really like lumpy. And my bra size was like tripled. My bra was double D. I was so happy when my milk dried up.”*	At 6 months she continued to breastfeed and had not used formula. Was feeding the baby solids at that time.	She states that “*I breastfeed for three weeks, but he wasn’t gaining enough weight. Because I would get like an ounce every time. So when the nurse come out she made, she put him on a bottle.*
				After she started using the bottle, the baby *“wouldn’t latch on so I just pumped and mixed”*

Notes for Table 1:

The follow-up interview for Teen 1 was accidently conducted too early, at week 7, when the mother was still pumping.

The follow-up interview for Teen 4 was conducted at 6 months, to reduce the possibility of loss to follow-up, even though the mother continued to breastfeed.

Teen 5 used the term “breastfeeding” to refer to pumping, which is not an uncommon practice among the teens in this study. We know she was pumping because of the weekly tracking data.

“I was learning in the classes that it’s better milk. They have more – they have less ear infections, um, less breathing problems, you know? More healthy, more routine and stuff like that . . . I think it’s an excellent thing because the child has better qualities.”  (Teen 2).

“I wanted to do it just because, like they say, it makes them not get as sick as much, infections and all that. And he’ll be smarter. Just healthier, I guess. So, I didn’t have to have my period. And because they say you lose your weight faster.”  (Teen 5).

Teen 4 breastfed for a different set of reasons starting with a strong determination that stemmed from her opposition to formula: *“I don’t want to go to formula. I don’t want my baby to even know what formula is…I don’t even want to be in the state’s system. The only thing I want to be in the state system for is Medicaid . . . because I can’t pay for Medicaid . . . My main reason [for breastfeeding] was less money I had to spend. Like I said, I don’t have the funds to get formula milk . . . Two, my mom did it . . . And then I’ve always heard from the TPMP, ‘breastfeed, breastfeed, breastfeed, breastfeed, breastfeed, breastfeed, breastfeed.”’* Her other reasons include financial constraints, her need for financial independence, her mother’s positive breastfeeding experience, and the encouragement of the TPMP.

All five teens who intended to breastfeed did initiate; however duration was shorter than might be expected from their prenatal intentions with only one exception (Teen 4) (see Table 1). Milk expression by pumping was an integral part of their breastfeeding practices and extended the duration of human milk feeding. Four of the five breastfed for similarly short durations; these four mothers collectively fed at the breast for 9 days. The addition of pumping to breastfeeding or pumping exclusively extended the provision of human milk to 109 days. Teen 4, in contrast, continued to breastfeed at 6 months at the time we conducted her follow-up interview; she exclusively breastfed until she started offering solid food at about 5 months.

#### Mixed breastfeeding messages and support

All of the teens had adults in their lives who were supportive of their breastfeeding. Two (Teens 1 and 4) had mothers who had breastfed; the mother of Teen 1 was breastfeeding her own baby at the time of the study. This same teen indicated at follow up that she received support from the nurse at school, a doctor and staff of the WIC (Women, Infants and Children) program at the health department. Teen 3 was told by the school counselor (at baseline) that she would help the teen to pump at lunch and she could *“leave class—my last class 5 minutes early before the buses get here, to pump”.* Teens 3 and 5 indicated that *“everybody”* wants them to breastfeed because it is best for the baby*.* Teen 4’s mother had breastfed and she brought the baby to campus so the teen could breastfeed during the day: “*I was away from the baby for a week [for college]. And they had programs back-to-back so I wasn’t able to go home. My mom was coming to [campus], getting the breast milk I was pumping, and taking it home. At that time I only had a hand pumpa. . . so while pumping the other one is leaking. . .So my mom would come . . . and I’d feed her . . . I give [my mom] the milk I have.”*

The teens noted that their friends offered more negative views of breastfeeding. Teen 1 for example reported that she received only negative comments from her friend: *“Some people told me, like my friend at school, “Why you breastfeeding, your, you know, it will get uglier.”* [Interviewer: How do you feel about what they said?]. *“I don’t feel bad at all. But what they say is still opinion, you know. But I don’t think that way.* Teen 2 indicated at baseline that she had not told her boyfriend she planned to breastfeed and Teen 4 reported that her boyfriend told her that “*breastfeeding is not for the baby – I mean breasts are not for the baby. But for. . . [pause *][Interviewer: He’s thinking sexual?] “*Yes. But he says it’s cheaper too *.” The latter was a key reason why Teen 4 breastfed, as previously mentioned.

Teen 4 learned a different message from the infant feeding experiences of those around her: “*I don’t want to go to formula. I don’t want my baby to even know what formula is. . . Friends I’ve had – like a friend I’ve lived with she had a baby not too long ago. And her baby was going to the hospital every other week, and were like ‘What is wrong with her’. And she’s like ‘Oh, it’s her formula is breaking her out. Oh her formula is messing up her stomach’. She had to change her formula so many times.”* This teen also received good support from her mother who brought her baby to campus during the day so she could breastfeed.

#### Poor breastfeeding knowledge and skills

Although all the teens in the study had received basic breastfeeding education as part of their childbirth class, they started their breastfeeding journey with little knowledge about the challenges of breastfeeding and without the skills needed to sustain it. While many of the teens indicated having “support” for their decision to breastfeed, this support was not accompanied by knowledge that could help them breastfeed or solve problems they encountered. Except for the two teens whose mothers had breastfed, the teens themselves and those in their social networks appeared to be uninformed about how to breastfeed. Unfortunately, these young women “don’t know what they don’t know.” When asked at follow-up interviews about information that they wished that they had known about breastfeeding, three of the four teens who had multiple problems stated they knew what they needed to know about breastfeeding. Mostly, they seemed to recall information about the different positions they could use, but they did not have the knowledge or skills needed to be clear about things that were going wrong or actions that they could do differently to solve breastfeeding problems: *“I know what positions I should put him in. Well, the best position is the football position. And that’s all I needed to know. And like how to, uh, how to store my milk and stuff like that”* (Teen 2). Teen 3 indicated at follow-up that she wished she had known more about the difficulties of breastfeeding and had more information about how to continue breastfeeding without stopping out of frustration. She stated that she would like for others to know *“It’s not going to always work the first time. You have to keep going and keep going for it to get easier. It’s going to get harder before it gets easier I should say” *.

Teen 4, in contrast, received good advice from her mother who breastfed, as exemplified in this quotation: *“She taught me how to cover my breast to cover up the right way. Because I used to have all this showing. . . Which is really good. Because. . .there was one time I was on a city bus, and I had to feed her. And I was trying to hold off. I was like, “Come on. Come on. We have 15 minutes.” And I knew I couldn’t wait 15 minutes, but I was trying. . . And the people started looking at me. I was like, “You know what?” And people, just some old women who were riding, “Oh, it’s so cute.” You know, other people were like, (laughing). I was like making sure I was covered, so.”*

Teen 4 was the only mother in the study to breastfeed long enough to perceive any benefits of breastfeeding. At follow-up she described her belief that breast milk stimulated the baby’s development: *“Right now the baby’s smart. She’s growing, too. She knows to turn to sound. Even though you’re supposed to be doing it now anyway. She was doing it a month before she was supposed to. Like most babies I know, they couldn’t push off their feet at four months. But they weren’t trying to scoot at four months, they don’t start to six months. She’s trying to stand up on her own. Her arms need to get a little stronger. . . Her legs are strong enough. And I’m like if I had done formula like the rest of them, my baby wouldn’t even walk. . . The two-year-old I was telling you about, she didn’t walk until her first [birthday]. . . And those examples, it was like, OK, yeah, I’m breastfeeding. Both of them did formula.”*

#### Uncontrollable and unpleasant physical experience with breastfeeding

Perhaps in part because of their own and other’s lack of knowledge and skill, breastfeeding was more complicated than they either expected or could manage, and they quickly felt overwhelmed and out of control. Their stories indicated that they could not tell when they were getting into trouble with breastfeeding and did not have anyone who could help them problem-solve. In these situations they lacked the confidence and skills needed to take control over their own breastfeeding practice. Instead, they let the baby or their breasts control the feeding practice. The result was that the breastfeeding experience for four of the five young mothers was very physical and unpleasant. Unfortunately problems common to many women, such as pain and leaking, and their poor ability to manage them, created significant challenges for many of the mothers and contributed to their unpleasant experiences. Teen 1 breastfed for 1 week, but she stopped because she did not like *“putting the baby to the breast. . . it feels funny, it tickles.”* She did continue to exclusively pump until she went back to school, when she added formula to her pumping practice. The teen indicated that she did this because *“Sometimes I have to get all wet and have to cover up so people don’t see. That’s a problem.”* Teen 2 stated ‘*When new milk comes it hurt, really bad. . . That’s like where they couldn’t do nothing about that. Because if I put ice or cold water, it would dry up the milk, then he won’t have no milk. So that’s why I couldn’t do it.”* She decided to quit because her *“breasts felt like hard rocks,” and “Breast milk is better than formula milk, but I had to stop.”* After Teen 5 introduced formula (week 1) she experienced new problems with latching, which resulted in her pumping and mixing formula to feed the baby: *“He wouldn’t latch on. So, I just pumped and mixed it”* (continued pumping until day 28). Teen 3 experienced numerous problems during her short breastfeeding experience.

“I did breastfeed for like the first week, but she was – like I don’t know exactly what it was. She was kind of small, and like I just woke up, and I was just really big overnight. So, you know what I’m saying, I guess it kind of intimidated her. Because the day before like when I was in the hospital Thursday, which at the size where she could latch on—and be comfortable. But then when I woke up Friday and I tried. [Then my milk came in] and, it was just really big, and I was leaking, and like she would latch on. And, then, I guess it would come out too fast because she’s not used to that or something that she will pull away. And, then, she would latch on, pull away, latch on, pull away. And it got to the point where my skin was peeling off. It was just really painful . . . then I tried to give her formula during the night, and then breastfeed her during the day because with the breast milk she would wake up more often, because it’s not as heavy as the formula. So, she would get hungry faster. And I just got used to giving her formula, because it’s a lot easier. You just heat it up and give it to her. And, then, with me breastfeeding it was a lot harder anyway because I would have to pump in between . . . Because I was so full I would have to pump to relieve because they would be so hard and full. And I was leaking a lot . . . Just I couldn’t leave, like go nowhere, because I would have to feed her. . . So, I just like got in the habit of giving her formula.”

In contrast to the other teens, Teen 4 did not report an unpleasant experience and was able to exercise more control over her breastfeeding practice. She was able to sustain breastfeeding even when the baby was sick or ate poorly, and when she had sore nipples. She stated during the infant feeding tracking that although she did have sore nipples, this pain was not enough to cause her to quit breastfeeding. Her mother helped her with this by suggesting she use beeswax as a home remedy for sore nipples.

#### Inadequate health care response

The teens’ lack of knowledge about infant development and care as well as breastfeeding, increased their reliance on others, including health care providers. Unfortunately, the health care system was less than helpful. Despite the teens’ intentions to breastfeed, health care providers made statements and acted in a manner that led two of the teens to believe that formula was better for their babies. Teen 2 returned to the hospital at four days postpartum because she had high blood pressure. At that time the nurse advised her to give the baby formula because he *“wasn’t getting enough, he was crying because he was hungry”;* her story indicated that she might have had difficulty breastfeeding because she was engorged.

Teen 5 was also encouraged by health care providers to introduce formula. During the weekly tracking conversations, Teen 5 stated that she did not have any major problems, although she also stated *“If crying a lot, I put him to my boob and he falls asleep.”* Her baby’s weight dropped from 7 lbs 7 oz [3.37 kg] to 6 lbs 1 oz [2.75 kg] before a nurse initiated formula use during a home visit (probably three days postpartum). During her follow-up interview she stated: *“I didn’t know I wasn’t giving him enough milk because he wasn’t like he was getting any bigger. Like he only grew like two ounces since he left the hospital. When [the nurse] came to my house to do the little check-up she weighed him. And she was like, “Let’s just try to see if he gets any bigger just doing one bottle.” And she fed him a bottle, and he gained two ounces just like that. So, I didn’t ask nobody. I thought it was OK. But she was like, “It’s just best to put him on the bottle.”*

After the nurse introduced the formula, she experienced new problems with latching, which resulted in her pumping and mixing formula to feed the baby: *“He wouldn’t latch on. So, I just pumped and mixed it”.*

#### Breastfeeding cessation

The reasons the teens offered for why they stopped breastfeeding and/or pumping followed rationally from their experience of unpleasant and/or uncontrollable breastfeeding and health care recommendations to incorporate formula (see Table 1).

### Themes from life journeys

#### Hopes and expectations

Future expectations for all five teens included going to college, making their parents and children proud of them, and helping their children have a good life. All of them were able to remain in high school during and after pregnancy with the support of the “homebound” program that allowed them to keep up with classes at home. At follow-up three were back in school, one was on summer break, and one was in college. (See Table 2 for more detail on these aspects of the teens’ journeys.) As Teen 1 expressed it: “*I hope like next year she will be getting better, like growing up good. And I hope my score in school will be getting higher. Yeah, I want to be a good mom and a good student . . . And be successful”.*

**Table 2:** Elements of the Teens’ Life Journeys

**Teen 1**	**Teen 2**	**Teen 3**	**Teen 4**	**Teen 5**
**PERSONAL GOALS AND CONCERNSAT BASELINE**				
She hopes to make her parents proud, finish school, go to college and become a nurse. She wants to be a good mother and she also hopes to be married to her boyfriend so that he can take care of her, her family, and the baby: *“I just want to get married because I want him to be beside me and be there for me and my baby taking care of us and stuff… He want to be a good father and want to take care of my baby and my family and me.” *	She does not want any more children until she is 30, or at least after her 3^rd^ year in college. She desires to be a cosmetologist and a fashion designer.	She plans to complete high school, study business at the community college and provide for the baby in the best way possible.	She wants to go to college for the arts and possibly marry the baby’s father. If she doesn’t marry the baby’s father, she wants to find someone that will “love her and her baby.”	She wants to go to college after completing high school. She mentioned that she wanted to be a pediatrician or an attorney, but she has not decided.
She reported during the baseline interview that she felt she was too young to be a mother.	She is excited about becoming a mom, but her current concerns center around raising a boy: “*I was just talking to my, uh, my, uh, my mentor about the way I’m just nervous about having a boy… I really wanted to have a girl, because I thought I could relate to her better, you know? But I didn’t think I could really raise a boy, but since I now I know I’m having a boy, I’ve got to deal with it *.” She does not want the child to end up in juvenile facilities or to do the same things that she used to do. She wants her baby to “*be civilized and a good person.”*	At baseline she did not report any concerns except “delivery”.	She reported at baseline*, “They say ‘Babies are a blessing’, but what, I mean, I’m not saying my baby was a mistake, but it wasn’t on purpose either. So I just hope she doesn’t end up like me…getting pregnant. I want her to be able to do her plans. I was like, ‘I’m going to college. I’m getting out of high school, going to college. Get married first’, you know, all that good stuff.*	
			*Now I have to change my life. Okay, baby here. Go to college. Maybe get married to the baby’s father.*	
**LIFE SITUATION AT FOLLOW-UP**				
She is living with her mom, dad, brother and sister. The baby’s father married the teen, and has also moved into the home. The teen’s family and the baby’s father help her with the baby. When she goes to school, the baby “*goes to day care*.” She mentioned that she gets help from the nurse at school, but she did not specify the kind of help that she receives. Her grandmother and sister watch the baby when she goes out with the baby’s father and when she does her homework. She believes she gets support from her family *“Like when I get tired and stuff they take care of my baby. . .sometime I need a break. Me and my husband go watch a movie”. . . .I’m okay [with being a mother] because, you know, after school I get to spent time with her and I got time to do my homework. That gives me a little time because I’ve got support from my family. They can take care of her while I do my homework and all the stuff.*	She is living with her mother and siblings and was preparing to live with the baby’s father at the time of the follow-up interview. Of her life she states, “*It’s been 2 months since I had my baby. Now I work at least about 50-something, 40-something hours at my job. I still hang with my friends and stuff, and I spend a lot of time with my boyfriend. He helps with the baby a very lot. I put him in daycare this week. My mom keeps him when I go to work. Together at home, we just like just sit up and watch TV. I’ll play with him. And like he’ll want a bottle or something, and I’ll feed him, and he’ll sleep like half the day, and half the day he’ll be up. “*	She has not yet gone back to school. She currently lives with her mom, sisters, brother and grandmother. Her boyfriend *“Is learning slowly but surely”* [about helping with the baby]. Her mother just had a baby: *“it’s kind of a pro and a con. Because it’s a pro because she can help me and I can help her…But it’s a con because it’s like two babies”.* Her grandmother and boyfriend’s aunt will help with the baby while she is in school. She gets support from her TPMP mentor *“I text her all the time…Even if it is not about the baby. She just checks up on me. Like we’re friends.” …She told me that if I ever felt overwhelmed or if I just need like ‘oh, my goodness, I need somebody t otalk to or something’ she said I could call her.”*	She is a freshman in a college, which stipulates: she must live in the dorm; she cannot bring her baby to her dorm room; and she must spend the weeknights at her room: *“My residence director told me “don’t bring her here, because if she falls off the bed, she get hurt or something. I’m like ‘she doesn’t even move. She’s going to sit right there.’ I can see if she’s one years old running around. But they were like ‘to make it fair to everyone no matter how old your baby is she can’t come’. And I was like ok. But she can stay in the parlor ifshe’s with me.”* She goes to her mother’s house after school and then takes the bus back to campus to be there by 11pm curfew. Various family members and the baby’s father help during the day and her own mothers keep the baby at night.	She currently lives with her mom and sisters and brother. Her grandmother watches the baby when she is in school. The teen’s father helps when he can, but he lives across town and does not have a job.
	She puts her baby in daycare and her mother also cares for him when she works. She believes her extended family is supportive *“Like my mom, she helps – she’ll keep the baby while I work or my boyfriend would, or his mom. And like if I need somebody like if I got a lot of things in my hand or something, I’ll say “can you grab that bottle. . . And like financially, um, my boyfriend, my mother, and his mother buy stuff. And that’s about it.”* She also gets support from her mentor at the TPMP.			
**MOTHERHOOD EXPRIENCES AT FOLLOW-UP**				
At 9 weeks postpartum, she said *“I don’t feel like I’m young, I feel like I’m getting old. I feel like I’m a mother, a woman… And I like it.”* Her hardest struggles included the baby’s crying and not getting sleep*: “Because you in school, you know, at night when the baby cry you don’t get much sleep. When you go to school, you don’t real concentrate on the school, because you get tired.”*	She described changes in her lifestyle since becoming a mother as *“I don’t party as much as I used to, I don’t do drugs, I don’t hang out with the wrong people.”* Current struggles include financial constraints and the necessity to keep the baby with her: *“I really want to get him circumcised and I don’t have moneyfor it. Or if I want to go somewhere and I don’t want to bring him, that’s a struggle I have…I have a baby now. I’ve got to bring him or I’ve got to stay at home and can’t go anywhere.*	After the birth, she spends most of her time with her baby and the father: *“Since I had he rI really try not to associate with a bunch of people…I think once you have kids your mindset kinda changes, and you…don’t want to hang around and do stuff you used to do as much…So I spend most of my time with my boyfriend or her [the baby].”* At four weeks postpartum she stated, “*My life changed a lot… Pretty much in good ways. Because I find myself, like I said, not wanting to like do things tha tI know that shouldn’t be doing. I always like think before I do something. It makes you a lot more cautious in what you do. I just – I just look at her, and I’m just happy that, you know what I’m saying, even though me being such a young age, I think that it’s made me mature and grow up, which I needed to do (laughing). It’s not easy, but I think after it’s all said and done it’s worth it.”*	Most of her struggles after becoming a mother stem from limited finances due to not being able to work, being in college, conflictual family relations and perceived dwindling of personal support and relationship with the baby’s father. She has food stamps and Medicaid, but she explained that her mom is “*going through hard times too*” and the family needs money. She is concerned about filing child support papers because of the perception that this action would put more strain on the relationship with the baby’s father. She also struggles with the limited time that she has with the baby: “*It’s unhelpful to me I always don’t spend as much time as I want to with her.*” She recognizes the trade-offs as she is wistful about missing programs at her college in order to spend time with the baby every day. She likes to have the baby sleep with her and see the baby smile when she wakes up. “*I – I just want to be a good example for my baby. I just – I want her to be able to be proud of me. Some people who have teenage mom, they’re not – it’s like they’re so ashamed of their mom or something. I don’t want that. I want her to be so – so proud. I want her to be able to, ‘My mom did this,’ and ‘My mom did that.’ I want her to brag. Not brag till people want to slap her. I just want – I just want her to be proud*” and “*So, all this going through this is like, ‘Why did I choose to keep her?’ but when I look at her, I – I see why I chose to keep her. [My baby] can have apple juice. So, I buy her apple juice, because I’m pumping as much milk as she eats. I can’t pump that much every two hours if I’m away from her for 12 hours. That means I’m pumping every two hours. But I’m only pumping two ounces. She eats six ounces every time she eats. So, just give her apple juice. Give her soup. Let’s get her food. Don’t be lazy and just put a bottle in her mouth. Feed her food [My family won’t] do that. And I’m like, ‘Please, feed her food. She’s not going to have anything to eat’ They have formula in there for back up. She doesn’t need formula for back up. Y’all might as well throw that away, because it’s going to get old. Don’t feed her formula. Feed her food. And they’re so lazy they don’t want to do it.*	She said: “*We hardly go out unless we’re going out to lunch or something…“Everybody is supportive, spending time with us and taking us where we got to go. Because I don’t have a car. I don’t got a job. So, I rely on somebody all the time. I mean I don’t get frustrated, but he does like - He stays awake all the time. It’s a struggle having to rely on everybody for everything. I want him in daycare so I can get a car and a job so I can do it on my own and not have to rely on everybody else. But I just love being a mom. Just brighten my world. It changed a lot, but it was for the better.*
As far as she knows she is the only teen her age to have a baby in her community.		The biggest struggle she mentioned is not getting enough sleep.		

#### Navigating multiple roles

The teens’ new maternal role was complicated by their other important roles as students, employed workers, and daughters. Teen 4 describes the challenges she faced integrating motherhood with being a college freshman and the tradeoffs she had to make trying to “juggle” her multiple roles *“I’m sitting here thinking, like if I was such a bad parent [as family members often tell her] I wouldn’t come out here [back home] every day even when I wanted to do program at my school. Because my college has a lot of things you can do. They always have a program going on. Now, I say, I really want to go there. But I sacrifice not going, because I know I have a baby. So, instead of staying at school, I come home every day even though sometimes I don’t want to.” *

Others also found combining motherhood with high school, employment and/or family obligations challenging:

“When I go back to school and I’m still breastfeeding it’s going to be hard also, because I’m still be producing milk, but I can’t relieve it how – like how I normally do. Because, then, I would have to pump during lunch. But I don’t think I could go for that long.” (Teen 3)

It’s been 2 months since I had my baby. Now I work at least about 50-something, 40-something hours at my job. I still hang with my friends and stuff, and I spend a lot of time with my boyfriend. He helps with the baby a very lot. I put him in daycare this week. My mom keeps him when I go to work. Together at home, we just like just sit up and watch TV. I’ll play with him. And like he’ll want a bottle or something, and I’ll feed him, and he’ll sleep like half the day, and half the day he’ll be up.” (Teen 2)

#### Youth and dependency

Four of the five teens in the study were living with their parents, and other family members, on whom they still depended. Their dependent status both complicates and eases their abilities to navigate their multiple roles since they have few financial or other resources and are typically dependent upon others for transportation, sustenance, and support. Such dependency increases the salience of the opinions and expectations of their extended family. For example, Teen 4’s mother banished her from the house when she was pregnant, but later provided her with extensive support, without which the teen would not have been able to breastfeed or manage college life. The others also relied heavily on their immediate and extended family members to care for their babies as well as provide economically for both mother and baby. Teen 3, whose mother had recently had a baby expressed it this way: *“It’s kind of a pro and a con . . . It’s a pro because she can help me and I can help her . . . But it’s a con because it’s like two babies”.* Both her grandmother and her boyfriends’ aunt helped care for the baby while she was in school. The teens also received financial support from the family networks: “*And like financially, um, my boyfriend, my mother, and his mother buy stuff.” * (Teen 2).

Dependency based on youth had its downside however. Teen 5 was very frustrated with not having a car and having to rely on everyone all the time:

“Everybody is supportive, spending time with us and taking us where we got to go. Because I don’t have a car. I don’t got a job. So, I rely on somebody all the time. . . It’s a struggle having to rely on everybody for everything. I want him in daycare so I can get a car and a job so I can do it on my own and not have to rely on everybody else. But I just love being a mom. Just brighten my world. It changed a lot, but it was for the better.”

“If I want to go somewhere and I don’t want to bring him, that’s a struggle I have. . . I have a baby now. I’ve got to bring him or I’ve got to stay at home and can’t go anywhere.” (Teen 2). 

Teen 4 struggled with her mother over the teen’s opposition to taking the legal steps necessary to receive child support. The teen’s mother wanted her to do this because they were struggling financially. The teen however did not want to do this because it would increase her dependency on the state, the baby’s father, and his father; this dependency would strain an already troubled and difficult relationship.

## Discussion

This qualitative prospective study explored the breastfeeding experiences of teen mothers during the early postpartum period. Because the breastfeeding duration for these young women was so short, the time period covered in this study, with the exception of Teen 4, was within the first eight weeks postpartum. DeVito’s qualitative study of the meaning and experience of teen mothers during this early postpartum period revealed that most are unprepared for the demands of motherhood and the changes in their life that it demands [22]. They often vacillate between wanting to mother and be mothered, suffer from serious sleep deprivation, and are unsure about what questions to even ask health care providers. Similar ideas emerged in the experiences of the mothers in this study and breastfeeding added to their uncertainty and confusion about motherhood.

For these young women, breastfeeding practices and cessation were an integral part of their experiences as a new mother in the context of their ongoing multiple roles, their living situations, their youth and dependency, and poor knowledge of the fundamentals of breastfeeding and infant development. The decision to stop breastfeeding was influenced by inadequate breastfeeding skill, a physically unpleasant and painful early experience they were unprepared to manage, and inadequate health care responses to real problems. Our findings parallel those of Wambach and Cohen who also found that early breastfeeding problems, such as leaking and pain, contributed to early weaning [23]. Unfortunately, the negative experiences overshadowed the potentially emotionally positive aspects of breastfeeding that other research has found to contribute to sustained breastfeeding [24].

Most teens lived with multiple immediate and/or extended family members upon whom they were dependent for support; consequently the expectations and beliefs held by these family members were salient. Their stories indicated that family relationships may be complicated; yet as Teen 4’s story illustrates, the importance of receiving instrumental support from family members remains critical despite having, at times, intense conflict with them. These experiences support the view of others who have found that social support is crucial for mothers to have after their initial decision to breastfeed and during the stages of learning how to breastfeed and adjusting to the process [24]. These young mothers, as with new mothers of all ages, need support in their roles as mothers and students, in addition to support for breastfeeding. Having support, however, was insufficient to sustain breastfeeding. All of the teens in our study indicated that they had adults around them who supported breastfeeding. Our findings suggest that the concept of breastfeeding support is multidimensional and that teens need practical and informational support as well as emotional support.

Our results indicate that teen mothers understand the public health message that breastfeeding is the healthier choice. While this belief may motivate young women to initiate, or “try” breastfeeding, it is not adequate to sustain it. Teen 4’s experience suggests that, in addition to educating pregnant teens about the health benefits of breastfeeding, educators may want to focus attention on the economics of infant feeding options and on the benefits and consequences of both breastfeeding and infant formula. Our findings are consistent with others that find comfort with formula feeding to be predictive of shorter breastfeeding [25]. Our study, similar to findings from studies of older mothers living in poverty, finds that weaning in the first weeks is mainly due to problems associated with exhaustion, breastfeeding problems such as pain, leaking, engorgement and cracked nipples [26]. Our findings suggest that it is important for teen mothers to have a positive physical experience with breastfeeding after leaving the hospital. Lactation specialists and other health care providers need to be upfront with teens about the potential problems, and teach them and their significant others how to manage common situations, especially engorgement, pain, and leaking, and strategize with them about how they can get affordable help once they are at home.

Unfortunately, our data suggest that health care providers may be quick to introduce formula to solve problems without appropriate recognition of the teen mothers’ intentions to breastfeed. Our study did not allow us to examine these situations from the providers’ perspectives, who may have been appropriately concerned about the babies’ health and/or did not want the mothers to experience pain; nonetheless, the women’s stories suggest that the providers took infant feeding decision-making control away from the teens, rather than helping them navigate delicate situations in ways that might allow them to continue breastfeeding. Unfortunately, this loss of control feeds into the uncertainty most of these young mothers have about their own abilities to resolve breastfeeding-related problems or mother their babies correctly. When faced with situations they do not understand or know how to control (i.e., pain; leaking; poor latch; perceived hunger; and baby’s weight loss), most of them concluded that the “baby” did not like breastfeeding or that formula was necessary. It is important for teen mothers to feel competent, so rather than take control away, we recommend that providers and educators work with teen mothers to make informed decisions and learn the skills needed to manage common problems and the skills that can increase their sense of agency and efficacy as mothers [27].

Consistent with DiVito’s findings, we found that most teen mothers “don’t know what they don’t know” and may believe, incorrectly, that they learned everything they needed to learn from a basic breastfeeding class [23]. This suggests that it is important for health providers and support persons to ask the teens about their concerns in specific, concrete terms, using strategies that help the teens voice their concerns. Prenatal education needs to be grounded in helping teens understand not only the basics of breastfeeding management, but also important factors related to normal infant development, including weight gain and loss, sleeping, crying and eating patterns, and infants’ emotional needs. The importance of significant others in the teens’ lives suggests that educational programs may want to encourage the teens to bring along people on whom they will be relying for day-to-day support.

For the teens in our study, milk expression by pumping was an important part of their feeding practices. The teens introduced pumping into their daily routines soon after discharge from the hospital, even though they did not have to return to school for some weeks later. This is a complicated issue since pumping in the early weeks can make the early breastfeeding experience more complicated and time consuming, and may decrease ability to produce sufficient quantities of milk in later weeks. Although it may well be very important for these mothers to learn how to use the pump successfully as part of their preparation for returning to school, they might have more breastfeeding success if they were to delay pumping until four to six weeks postpartum, which for many is two weeks before returning to school, which for many is six weeks postpartum [28]. Therefore, it may be important to schedule a specific time after delivery to teach teen mothers how to properly use a pump or to hand express and to integrate this practice into their daily routine that includes feeding at the breast when together, but also milk expression when spending long hours away from the baby.

## Conclusion

The strengths of this study include its prospective study design and our combination of baseline and follow-up qualitative interviews with weekly infant-feeding tracking. Although our study is limited by the small sample size and having recruited from a single program, it offers an intimate view of the breastfeeding practices that may be reflective of a largely minority population of low-income teen mothers. These young women’s lives are more complicated than those of many mothers who have the advantage of greater experience, maturity and resources. Yet the positive experience of one young mother in this study, and her ability to exclusively breastfeed for six months despite a complicated life situation, indicates that these young mothers can breastfeed and continue their education if they have the determination, knowledge, skills, control, resources and support they need to have a high quality breastfeeding experience. This determination seems to depend on a complex interplay between different factors, including the having made an informed choice, and having the skills, support and experiences needed to sustain the belief that breastfeeding is the best choice for them and their baby given their life situation. It is important that they have a positive and enjoyable breastfeeding experience in the early days postpartum, that they are able to identify and claim a reliable support system that both supports their breastfeeding and can help them problem solve, and that the young women gain through their experience a belief in their own agency and competency as mothers.

## Abbreviations

WIC: Special Supplemental Nutrition Program for Women, Infants, and Children; YWCA: Young Women’s Christian Association; TPMP: Teen Parent Mentoring Program.

## Competing interests

The authors declare that they have no competing interests.

## Authors’ contributions

PHS conceived of and designed the study and the breastfeeding intervention and was involved in all phases of the study implementation, data analysis and manuscript writing. SLC conducted interviews and performed qualitative analyses and helped draft the manuscript. ML and EN participated in study and intervention design, data interpretation, and helped draft the manuscript. SC participated in study design, coordinated data collection and helped draft the manuscript. All authors read and approved the final manuscript.
